# Religious Coping and Diabetes Self-Care Behaviors Among Adults with Diabetes in Saudi Arabia: A Cross Sectional Study

**DOI:** 10.3390/healthcare14162440

**Published:** 2026-08-07

**Authors:** Alya Alghamdi, Majed M. Aljabri, Bandar S. Alharbi

**Affiliations:** Community and Psychiatric Mental Health Nursing Department, College of Nursing, King Saud University, Riyadh 12375, Saudi Arabia; amajad@ksu.edu.sa

**Keywords:** diabetes mellitus, diabetes self-care, religious coping, spirituality, self-management, Saudi Arabia, Brief RCOPE

## Abstract

**Background**: Religious coping is an important psychosocial resource that may influence how individuals adapt to chronic illnesses and engage in health-related behaviors. While studies have associated religion with the effective management of diabetes, the involvement of religious coping, both positive and negative, in the self-care of diabetic patients has been underexplored. The aim of this study was to quantify the association of religious coping with diabetes self-care among adults in Saudi Arabia. **Methods**: We used a cross-sectional study among 413 adults with diabetes. Positive and negative religious coping were assessed using the Brief Religious Coping Scale (Brief RCOPE), and diabetes self-care was measured using the Summary of Diabetes Self-Care Activities (SDSCA) questionnaire. Multivariable linear regression models were used to examine associations between religious coping and diabetes self-care after adjustment for sociodemographic and clinical characteristics. We also used quantile regression to assess whether associations varied across the distribution of diabetes self-care scores. **Results**: The finding showed that positive religious coping was significantly and positively associated with diabetes self-care (β = 0.44, 95% CI: 0.18 to 0.70), while negative religious coping did not show significant association (β = 0.04, 95% CI: −0.13 to 0.22). Quantile regression analyses demonstrated significant positive associations between positive religious coping and diabetes self-care at the 25th percentile (β = 0.41, 95% CI: 0.10 to 0.73), 50th percentile (β = 0.50, 95% CI: 0.11 to 0.89), and 75th percentile (β = 0.48, 95% CI: 0.03 to 0.92), with the strongest association observed at the 90th percentile (β = 0.95, 95% CI: 0.42 to 1.48). Negative religious coping was not significantly associated with self-care at any quantile. **Conclusions**: Our finding showed significant association between positive religious coping and diabetes self-care, whereas negative religious coping showed no significant association. The findings suggest that positive religious coping may represent an important psychosocial resource associated with diabetes self-care. Future longitudinal studies incorporating objective clinical outcomes are needed to further examine the role of religious coping in diabetes management.

## 1. Introduction

According to the global burden of diseases, diabetes mellitus remained one of the most common chronic diseases worldwide [1]. It represents a major public health challenge due to its high prevalence, long-term complications, and substantial healthcare burden [2]. Effective diabetes management depends heavily on patients’ engagement in self-care behaviors, including adherence to medication, healthy eating, regular physical activity, blood glucose monitoring, and foot care [3]. Engagement in behaviors is associated with improved glycemic control and a lower risk of diabetes-related complications [4].

Psychological and emotional stress are the main problems related to diagnosis and management of diabetes. Individuals with diabetes must cope with ongoing concerns related to disease progression, treatment adherence, complications, and lifestyle restrictions, and those can adversely affect self-management behaviors [5,6]. One study also suggested that coping strategies is an important determinants of adaptation to chronic illness and may influence patients’ ability to engage in effective self-care practices [7].

Among the various coping resources available to individuals with chronic diseases, religion and spirituality have received increasing attention in recent decades. Religion remains a central aspect of life for a large proportion of the global population and often influences health beliefs, decision-making processes, emotional responses, and health-related behaviors [8]. Several studies have shown that religious involvement is associated with improved mental health, greater resilience, lower levels of depression, enhanced quality of life, and better adaptation to chronic illness [8,9,10]. However, due to the nature of multidimensional construct of religion, its effects on health may depend on how individuals utilize religious beliefs and practices when confronted with stressful situations. Importantly, religious coping is distinct from general religiosity or spirituality because it focuses specifically on how religion is used during periods of adversity. Religious coping is commonly categorized into positive religious coping and negative religious coping [11]. Positive religious coping reflects adaptive strategies such as seeking spiritual support, collaborating with God in problem solving, finding meaning in suffering, and maintaining trust in divine guidance. In contrast, negative religious coping reflects spiritual struggle and may include feelings of abandonment by God, perceptions of divine punishment, religious doubt, or spiritual discontent [11,12].

Previous studies on the association between religion and diabetes has generally highlighted the importance of religious and spiritual resources. For example, one study among African American adults with type 2 diabetes found positive association between spiritual and religious beliefs and practices, and diabetes self-care activities [13]. Similarly, another study found that spirituality and religious resources contributed to coping and adaptation among individuals managing diabetes [14]. However, findings across studies remain heterogeneous, partly because many investigations have focused on broad measures of religiosity or spirituality rather than specific coping mechanisms.

Several important gaps remain in the literature. Most previous studies have examined religiosity, spirituality, or religious participation rather than positive and negative religious coping as distinct constructs. On the other hand, studies have often focused on psychological outcomes such as depression, distress, and quality of life rather than behavioral outcomes such as diabetes self-care. There are also only few studies that simultaneously examined both positive and negative religious coping within the same population. To our knowledge, evidence from Muslim populations remains limited despite the prominent role of religion in daily life and health-related decision-making within many Islamic societies.

Saudi Arabia provides a particularly important setting for investigating these relationships. The country has one of the highest diabetes prevalence rates globally and faces a substantial burden of diabetes-related complications [15]. At the same time, religion plays a central role in Saudi society and may substantially influence coping processes, health beliefs, and self-management behaviors. Although previous studies have suggested that religious and spiritual resources may support diabetes management [13,14], most have focused on general spirituality, religious beliefs, or religious practices rather than positive and negative religious coping as distinct constructs. Moreover, evidence from Muslim populations, particularly in Saudi Arabia, remains limited. Understanding whether positive and negative religious coping are associated with diabetes self-care may help identify culturally relevant psychosocial factors that can be incorporated into patient-centered diabetes education and behavioral interventions. Therefore, in this study we aimed to quantify the associations between positive religious coping, negative religious coping, and diabetes self-care among adults with diabetes in Saudi Arabia. We hypothesized that positive religious coping would be positively associated with diabetes self-care behaviors, whereas negative religious coping would be negatively associated with diabetes self-care behaviors. Additionally, we employed quantile regression analyses to investigate whether these associations differed across the distribution of diabetes self-care scores.

## 2. Methods

### 2.1. Study Design

A cross-sectional study design was used in this study. Data were collected across four primary healthcare centers in Riyadh, Saudi Arabia. The four primary healthcare centers were purposively selected to capture geographic and sociodemographic diversity across Riyadh. Centers serving different districts of the city were included to increase variation in the study population with respect to demographic and socioeconomic characteristics. The centers were selected to enhance the diversity of the sample rather than to provide a statistically representative sample of all primary healthcare centers in Riyadh or Saudi Arabia. The study was designed to evaluate the association between relatively stable psychosocial characteristics and diabetes self-care rather than temporal or seasonal variation. Therefore, recruitment over a two-week period was considered adequate to achieve the target sample size. An a priori sample size calculation was not performed because recruitment was based on the number of eligible patients attending the participating primary healthcare centers during the study period.

### 2.2. Participant and Sampling

In this study, the target population are adult patients (aged 18 years and above) attending primary healthcare centers in Riyadh who had a confirmed diagnosis of diabetes. Participants were recruited through two complementary channels. First, QR codes linked to the electronic questionnaire were posted on announcement boards within the waiting areas and patient-accessible areas of each participating healthcare center, allowing patients to access and complete the survey independently using their personal devices. Second, head nurses at each center assisted in identifying and approaching eligible patients during their scheduled visits, directing them to the survey link. Eligible participants were adults aged 18 years or older with a documented physician diagnosis of diabetes receiving care at the participating primary healthcare centers. For participants recruited through QR codes, eligibility instructions specified that only individuals with a prior physician diagnosis of diabetes should complete the questionnaire. Patients were excluded if they declined to participate or were cognitively unable to complete the survey. Data were screened for completeness before analysis. All questionnaires included in the analysis contained complete information for the variables of interest; therefore, no imputation procedures were required. Questionnaires were reviewed for completeness before analysis. Responses missing key study variables were excluded from the final analytical dataset.

### 2.3. Measures and Covariates

In this study, religious coping was measured using the Brief RCOPE, which is a widely used 14-item scale developed by Pargament and colleagues (Pargament, Koenig, & Perez, 2000) to assess positive and negative forms of religious coping in the context of stressful life events and health-related challenges [16]. The scale consists of two subscales: the Positive Religious Coping subscale (7 items; e.g., “Sought to strengthen my relationship with God,” “Tried to act while trusting in God”) and the Negative Religious Coping subscale (7 items; e.g., “Wondered whether God had abandoned me,” “Felt that God was punishing me for my sins”). Items are rated on a 4-point Likert scale ranging from 1 (Not at all) to 4 (A great deal). Subscale scores are computed as the mean of the respective items, with higher scores indicating greater use of the corresponding coping style. We used the Arabic version of the Brief RCOPE in this study. The Arabic adaptation has been validated and demonstrated acceptable psychometric properties in Arabic-speaking Muslim populations, with the subscales showing adequate internal consistency and construct validity [17,18].

Diabetes self-care behaviors were assessed using the Summary of Diabetes Self-Care Activities (SDSCA) scale, originally developed by Toobert, Hampson, and Glasgow (2000) [19] and subsequently revised to improve clinical utility and reduce participant burden [20,21]. The tool is a well-validated, multidimensional instrument that measures adherence to key self-care behaviors in patients with type 2 diabetes over the preceding seven days. In this study, four subscales were used: (1) Exercise (2 items: days with at least 30 min of physical activity; days with specific exercise such as walking); (2) Blood Glucose Monitoring (2 items: days blood glucose was tested; days glucose was tested as recommended by the physician); (3) Foot Care (2 items: days feet were inspected; days the inside of shoes were checked before wearing); and (4) Medication Taking (1 item: days diabetes medications were taken as prescribed). In addition, a total self-care score was computed as the mean across all subscale items. Each item is scored on a scale from 0 (none of the past 7 days) to 7 (all of the past 7 days), with higher scores indicating greater self-care engagement. The overall diabetes self-care score was calculated as the mean of the included SDSCA items, with higher scores indicating better diabetes self-care. The Arabic version of the SDSCA was used in this study. The Arabic translation and cross-cultural adaptation of the SDSCA has been validated and reported to demonstrate satisfactory reliability and structural validity among Arabic-speaking populations with diabetes [22,23].

Several sociodemographic and clinical profiles were also collected; including participants’ age (18–24, 25–34, 35–44, 45–54, 55–65, and >65 years), gender (male, female), employment status (employed, unemployed, retired, or student), marital status (married, single, widowed, or divorced), and educational attainment (below secondary, secondary, diploma, or bachelor’s degree or higher). Clinical variables included duration of diabetes diagnosis (<1 year, 1–5 years, 6–10 years, >10 years), type of treatment currently used (oral medications, insulin, combined oral medications and insulin, or diet only), presence of diabetes-related complications and their type (kidney problems, retinopathy, neuropathy, diabetic foot ulcer, other, or none), whether the participant works in a healthcare field, presence of other chronic diseases, and frequency of physician visits per year.

### 2.4. Reliability Check

The internal consistency reliability of the Brief Religious Coping Scale (Brief RCOPE) was assessed using Cronbach’s alpha coefficients. Reliability analyses were conducted separately for the Positive Religious Coping and Negative Religious Coping subscales. Cronbach’s alpha values ≥ 0.70 were considered indicative of acceptable internal consistency.

### 2.5. Statistical Analysis

Descriptive statistics were reported as means ± standard deviations for continuous variables and as frequencies and percentages for categorical variables. We also assessed bivariate associations between religious coping (positive and negative) and total diabetes self-care using correlation.

In this study, three linear regression were estimated to examine the association between religious coping and total self-care. Model 1 examined the unadjusted association between positive religious coping and self-care; Model 2 examined the unadjusted association between negative religious coping and self-care; and Model 3 was a fully adjusted model including both positive and negative religious coping as well as sociodemographic (age, gender, employment status, marital status, educational level) and clinical covariates (diabetes duration, healthcare occupation status, presence of chronic diseases, treatment type, presence of complications, and frequency of physician visits). Results were provided as regression coefficients (β) with corresponding 95% CI. All statistical tests were two-sided, and a *p*-value < 0.05 was considered statistically significant. We assessed the assumption of linear regression through residual diagnostic plots, including residuals versus fitted values, normal Q-Q plots, scale-location plots, and residuals versus leverage plots. Multicollinearity was evaluated using variance inflation factors. Multicollinearity among predictor variables was assessed using generalized variance inflation factors (GVIFs). For categorical variables with more than one degree of freedom, adjusted GVIF values (GVIF^(1/(2 × df))) were examined. Values below 5 were considered indicative of an absence of problematic multicollinearity.

To complement the results from multiple linear regression and to explore whether associations between religious coping and self-care differed across the distribution of self-care behaviors, quantile regression models were estimated at the 10th, 25th, 50th, 75th, and 90th percentiles. We incorporated the same set of covariates as the fully adjusted linear regression model. Bootstrap standard errors were used for inference in all quantile regression models. Standard errors for the quantile regression models were estimated using 1000 bootstrap replications.

All statistical analyses were performed in R version 4.4.2 [24] using the *stats* package (version 4.4.2), *car* (version 3.1.3), *quantreg* (version 6.1), *boot* (version 1.3.31), and *ggplot2* (version 4.0.1).

## 3. Results

A total of 413 adults with diabetes participated in the study. The largest age group was 45–54 years (29.3%), followed by 35–44 years (21.8%), while only 5.6% were aged 18–24 years (Table 1). Most of the participants were male (68.5%), and nearly half were married (48.9%), whereas 39.5% were single. More than half of the participants were employed (57.1%), while 21.3% were unemployed, 15.7% were retired, and 5.8% were students. Educational attainment was relatively high, with 41.2% reporting a bachelor’s degree or higher, whereas 19.1% had an education level below secondary school. One third of participants had been diagnosed with diabetes for 1–5 years (33.2%), and 31.0% had lived with the condition for more than 10 years. Most participants did not work in the healthcare sector (83.3%), and approximately two thirds reported having at least one chronic comorbidity (66.1%). Diabetes complications were reported by 26.9% of participants. Insulin therapy was the most commonly reported treatment modality (33.9%), followed by combined oral medication and insulin therapy (24.7%), oral medication alone (21.8%), and diet-only management (19.6%). About 48.9% reported at least three physician visits annually, including 29.8% who attended 3–4 visits per year and 19.1% who reported more than four visits per year.

The mean positive religious coping score was 3.72 ± 0.52. In contrast, the mean negative religious coping score was substantially lower (1.74 ± 0.91). The mean diabetes self-care score was 3.26 ± 1.44 days per week.

**Figure 1:**
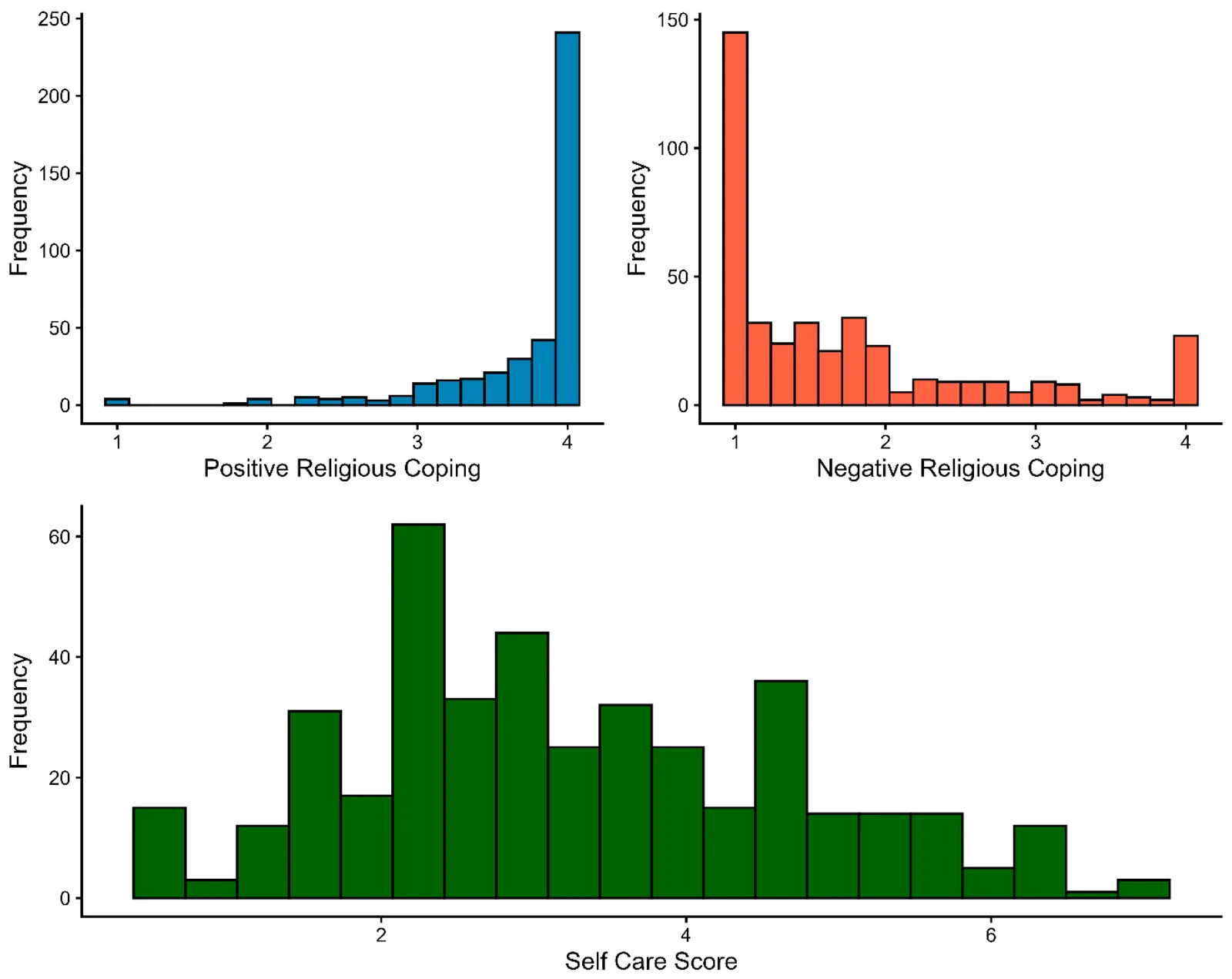
Distribution of positive religious coping, negative religious coping, and diabetes self-care scores among adults with diabetes.

Figure 1 presents the distribution of positive religious coping, negative religious coping, and diabetes self-care scores. It showed that positive religious coping scores were concentrated at the higher end of the scale. This indicates widespread use of positive religious coping strategies among participants. In contrast, negative religious coping scores were concentrated at the lower end of the scale. Diabetes self-care scores showed greater variability and were approximately normally distributed, which shows high heterogeneity in self-management behaviors across participants.

Correlation analysis showed a weak positive correlation between positive religious coping and overall diabetes self-care (r = 0.09), whereas negative religious coping was essentially unrelated to diabetes self-care (r = 0.01) (Supplementary Figure S1). Positive and negative religious coping were weakly inversely correlated (r = −0.09). This suggests that the two constructs represent distinct dimensions of religious coping. As expected, the overall diabetes self-care score was strongly correlated with its individual domains, including blood glucose monitoring (r = 0.76), exercise (r = 0.74), foot care (r = 0.69), and medication adherence (r = 0.59). Moderate correlations were also observed among the self-care domains themselves (r = 0.24–0.63).

Both the positive religious coping and negative religious coping subscales demonstrated excellent internal consistency (Cronbach’s α = 0.92 for both scales), indicating high reliability of the Brief RCOPE in this study population (Supplementary Table S1). The summary results for the associations between religious coping and diabetes self-care among adults with diabetes were presented in Table 2. In unadjusted model, there was significant association between religious coping and self-care scores (β = 0.46, 95% CI: 0.22 to 0.70, *p* < 0.001). This shows, each one-unit increase in the positive religious coping score was associated with a 0.46-point increase in the overall diabetes self-care score. This association remained significant after adjustment for sociodemographic and clinical characteristics (β = 0.44, 95% CI: 0.18 to 0.70, *p* < 0.01), indicating that positive religious coping remained associated with better self-management behaviors. However, the magnitude was modest and no minimal clinically important difference was established for the SDSCA total score. In contrast, negative religious coping was not significantly associated with diabetes self-care in either the unadjusted model (β = 0.08, 95% CI: −0.09 to 0.25) or the fully adjusted model (β = 0.04, 95% CI: −0.13 to 0.22).

In the adjusted mode, participants receiving combined oral medication and insulin therapy demonstrated significantly higher self-care scores compared with those treated with oral medications alone (β = 0.58, 95% CI: 0.08 to 1.09). Higher educational attainment and more frequent physician visits were also associated with better self-care, although these relationships did not reach statistical significance. Participants with a bachelor’s degree or higher tended to report better self-care than those with below secondary education (β = 0.59, 95% CI: −0.04 to 1.23), though it did not reach statistical significance. Similarly, individuals reporting more than four physician visits annually tended to have higher self-care scores than those with fewer than one visit per year (β = 0.62, 95% CI: −0.03 to 1.26), albeit not significantly associated. No significant associations were observed for age, gender, employment status, marital status, duration of diabetes, healthcare employment, chronic disease status, or diabetes complications. The fully adjusted model explained approximately 31% of the variability in diabetes self-care scores (R^2^ = 0.31; adjusted R^2^ = 0.26), indicating moderate explanatory power.

We assessed model diagnosis to assess the assumptions of the multiple linear regression model (Supplementary Figure S2). The residuals versus fitted values plot showed no major systematic pattern, suggesting that the linearity assumption was reasonably satisfied. The Q-Q plot indicated slight deviations from normality at the lower and upper tails of the residual distribution, although residuals generally followed the expected normal distribution. The scale-location plot suggested modest heteroscedasticity, with a slight increase in residual variability at higher fitted values. The residuals versus leverage plot identified a small number of observations with relatively high leverage; however, none appeared to exert undue influence on the model estimates. Overall, the diagnostic plots indicated that the model assumptions were reasonably met, with only minor departures from normality and homoscedasticity. All adjusted generalized variance inflation factor values ranged from 1.06 to 1.53, well below commonly accepted thresholds for problematic multicollinearity. It indicates that multicollinearity was not a concern (Supplementary Table S3).

**Figure 2:**
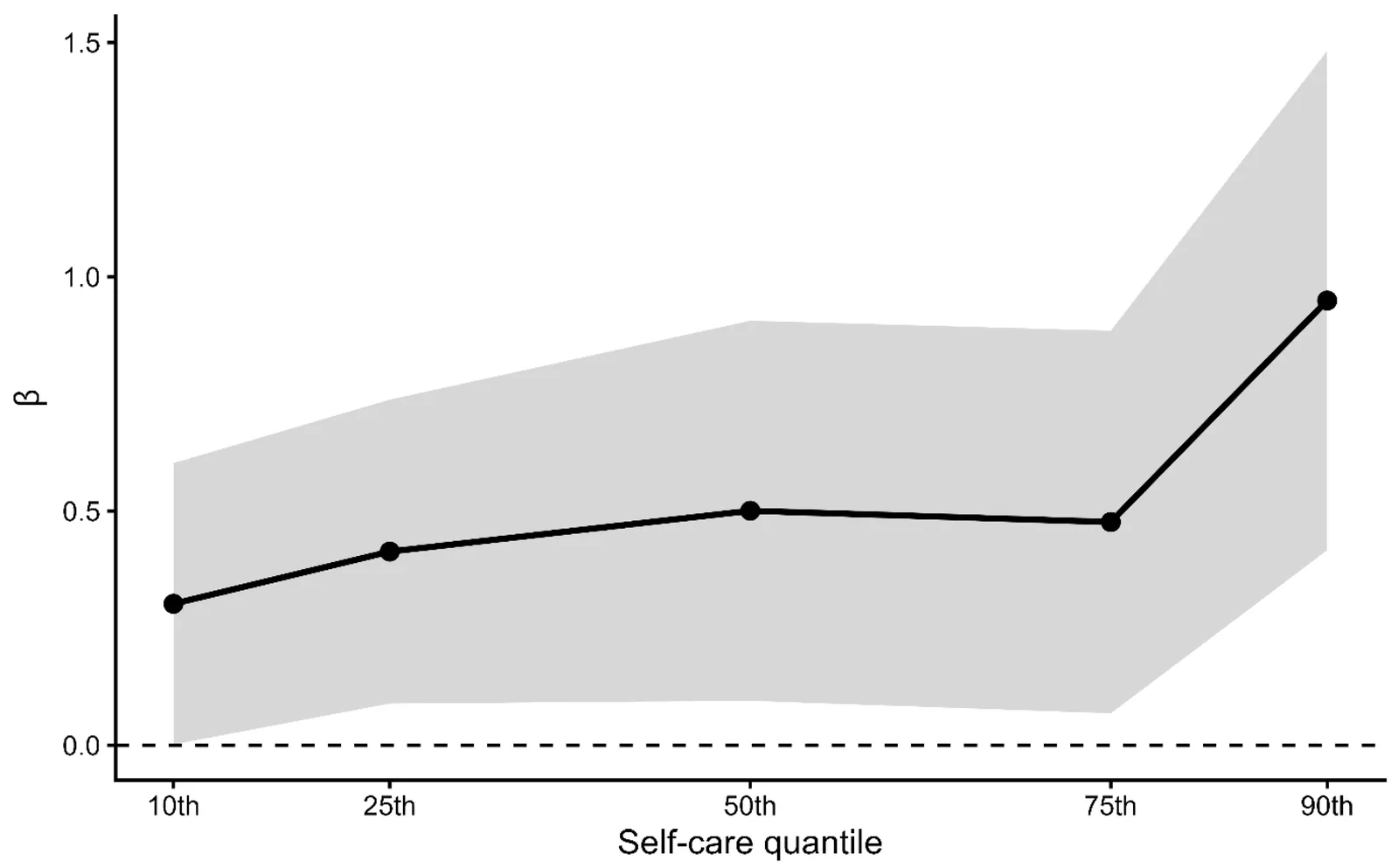
Quantile regression coefficients for the association between positive religious coping and diabetes self-care. Regression coefficients (β) and 95% confidence intervals are shown for the 10th, 25th, 50th, 75th, and 90th percentiles of the self-care distribution. Estimates were adjusted for sociodemographic and clinical covariates.

Quantile regression analyses largely supported the findings from the primary linear regression model. Positive religious coping was positively associated with diabetes self-care across multiple points of the self-care distribution, with significant associations observed at the 25th percentile (β = 0.41, 95% CI: 0.10 to 0.73), 50th percentile (β = 0.50, 95% CI: 0.11 to 0.89), and 75th percentile (β = 0.48, 95% CI: 0.03 to 0.92) (Supplementary Table S2). Although the association was weaker and did not reach statistical significance at the 10th percentile (β = 0.30, 95% CI: −0.02 to 0.62), the magnitude of the effect increased substantially at the 90th percentile (β = 0.95, 95% CI: 0.42 to 1.48) (Figure 2). In contrast, negative religious coping was not significantly associated with diabetes self-care at any quantile (Supplementary Table S2). As Figure 2 further illustrates, the positive association between religious coping and self-care became progressively stronger toward the upper end of the self-care distribution. This means that positive religious coping may be more strongly associated with self-care among individuals at higher self-care quantiles.

## 4. Discussion

### 4.1. Main Findings

In this study, we quantified the association between positive religious coping, negative religious coping, and diabetes self-care among adults with diabetes in Saudi Arabia. The findings showed that positive religious coping was significantly and positively associated with diabetes self-care scores. There was no significant association between negative religious coping diabetes and self-care. Quantile regression analyses further demonstrated that there was a significant association between positive religious coping and diabetes self-care across the distribution of self-care scores and appeared strongest among individuals with the highest levels of self-care. Our findings suggest that positive religious coping may represent an important psychosocial correlate of diabetes self-management in this population. Because participants were recruited from four purposively selected primary healthcare centers in Riyadh, the study sample may not fully represent the broader population of adults with diabetes in Saudi Arabia.

### 4.2. Comparison with Previous Studies

The positive association observed between positive religious coping and diabetes self-care is consistent with previous studies suggesting that religious and spiritual resources may support health-promoting behaviors and chronic disease management. Watkins et al. reported that spiritual and religious beliefs and practices were positively associated with several diabetes self-care activities among African American adults with type 2 diabetes, particularly dietary behaviors and foot care [13]. Although their study assessed spirituality and religious beliefs rather than religious coping specifically, both studies support the broader proposition that religious resources may be associated with engagement in diabetes self-management activities. Our findings are also consistent with the theoretical framework proposed by Pargament and colleagues, who conceptualized positive religious coping as an adaptive process involving spiritual support, collaborative problem-solving with God, benevolent religious appraisals, and the search for meaning during stressful experiences [25]. Positive religious coping has repeatedly been associated with improved adaptation to major life stressors and better psychological adjustment across a wide range of health conditions [26,27]. Although the association between positive religious coping and diabetes self-care was statistically significant, the observed effect size was modest and should therefore be interpreted with caution. A one-unit increase in positive religious coping was associated with an average 0.44-unit increase in the diabetes self-care score after adjustment for measured covariates. However, because no established minimal clinically important difference has been defined for the SDSCA, it is not possible to determine whether this magnitude represents a clinically meaningful improvement in diabetes self-care.

The association can be explained through a mechanism framework. It is possible that positive religious coping may be associated with better self-care through several potential mechanisms, including through greater optimism, perseverance, self-discipline, and perceived meaning in illness. Individuals who interpret illness within a framework of spiritual purpose may be more motivated to engage in behaviors that preserve health and adhere to treatment recommendations. Religious beliefs may also strengthen perceived social support through family networks, religious communities, and faith-based relationships, all of which have been linked to improved self-management behaviors [13]. Furthermore, positive religious coping has been associated with higher levels of psychological well-being and resilience. This may further facilitate sustained engagement in the daily tasks required for diabetes management [11]. However, these mechanisms were not directly measured in the present study and therefore should be regarded as plausible hypotheses derived from the previous literature rather than empirically demonstrated pathways. Future longitudinal studies incorporating mediation analyses are needed to determine whether these psychosocial factors explain the observed association.

In this study, the quantile regression findings showed that while the positive association between positive religious coping and self-care was observed throughout the distribution of self-care scores, the association became progressively stronger at higher quantiles. This pattern suggests that positive religious coping may be particularly relevant among individuals who are already highly engaged in self-management behaviors. One possible explanation is that positive religious coping reinforces existing health-promoting behaviors by strengthening motivation and persistence. Alternatively, individuals who consistently engage in self-care activities may experience greater perceived control and psychological well-being, which may coexist with stronger positive religious coping. Because of the cross-sectional design, these possibilities cannot be distinguished.

In contrast, we did not observe significant association between negative religious coping and diabetes self-care in either the primary or quantile regression analyses. This finding differs somewhat from previous studies showing that negative religious coping is associated with poorer psychological well-being, greater emotional distress, lower quality of life, and less favorable health outcomes [11,28,29]. Participants reported high levels of positive religious coping and relatively low levels of negative religious coping, a pattern that is consistent with the predominantly religious and culturally homogeneous study population. These distributions may reflect ceiling effects for positive religious coping and floor effects for negative religious coping, thereby restricting the variability available to distinguish individuals across the full range of the constructs. Several explanations may be possible for the lack of association observed in our study. The low mean score and restricted variability of negative religious coping may have reduced the ability to detect an association and may reflect a floor effect. Consequently, the absence of a statistically significant association should be interpreted cautiously. It might also be due to the fact that negative religious coping may be more strongly related to psychological outcomes such as depression, anxiety, distress, and emotional adjustment than to behavioral outcomes such as diabetes self-care. The cultural context may be important. Saudi Arabia is characterized by a highly religious social environment in which religion frequently serves as a source of support, meaning, and social integration. Hence, negative religious coping may occur less frequently or may have weaker associations with health behaviors than in populations where spiritual struggle is more common.

Although the unadjusted correlation between positive religious coping and diabetes self-care was relatively weak, the association remained statistically significant after adjustment for sociodemographic and clinical characteristics. This apparent difference is not unexpected because the Pearson correlation reflects the crude relationship between two variables, whereas the multivariable regression model estimates the association after controlling for potential confounding variables. The adjusted findings therefore suggest that positive religious coping has an independent association with diabetes self-care beyond the effects of the measured covariates. Nevertheless, the magnitude of the association remained modest and should be interpreted accordingly.

### 4.3. Clinical and Public Health Implications

Our findings suggest that positive religious coping may represent a relevant psychosocial characteristic to consider when providing culturally sensitive diabetes care in religious settings. However, because of the cross-sectional nature of the study, the findings should not be interpreted as evidence that assessing or promoting positive religious coping will improve diabetes self-care. In societies where religion plays a prominent role in everyday life, understanding patients’ religious coping strategies may help healthcare professionals better appreciate the psychosocial resources available to patients. Previous work has suggested that incorporating patients’ religious and spiritual beliefs into patient-centered education and support programs may improve engagement and communication between patients and healthcare providers [30,31]. While our findings do not imply that religious coping should replace evidence-based diabetes management strategies, they suggest that positive religious coping may represent an additional resource that some patients draw upon when managing their illness.

### 4.4. Strengths and Limitations

This study has several strengths. To our knowledge, it is among the few studies to examine positive and negative religious coping simultaneously for association with diabetes self-care among adults with diabetes and one of the first conducted in Saudi Arabia. Both subscales demonstrated excellent internal consistency in the study population, supporting the reliability of the measures. The inclusion of a relatively large sample of adults with diabetes allowed adjustment for a broad range of sociodemographic and clinical characteristics, reducing the likelihood that the observed associations were attributable to major measured confounders. The use of multiple linear regression and quantile regression analyses to quantify the association. We observed consistent findings.

Several limitations should also be considered when interpreting the findings. Since our study was based on cross-sectional design, it is not possible to conclude about the temporal trends, and it is not possible to assess the causality. This means, although positive religious coping was associated with higher self-care scores, it is not possible to determine whether religious coping influenced self-care behaviors, whether individuals with better self-care were more likely to engage in positive religious coping, or whether both were influenced by other unmeasured factors. Although recruitment was completed over a relatively short two-week period, the study focused on stable psychosocial characteristics rather than seasonal changes in diabetes self-care. Nevertheless, clinic attendance patterns, religious events, or other temporal factors during the recruitment period may have influenced participant participation and may limit the representativeness of the sample. All study variables were assessed through self-report questionnaires, which introduces the possibility of recall bias and social desirability bias. This issue may be particularly relevant for measures of religious coping and diabetes self-care because participants may have been inclined to report socially desirable behaviors or attitudes. Although we adjusted for a range of sociodemographic and clinical characteristics, several important psychosocial and clinical factors that may confound the association between religious coping and diabetes self-care were not measured. These include diabetes knowledge, health literacy, diabetes distress, depression, anxiety, self-efficacy, social support, glycemic control (e.g., HbA1c), and healthcare accessibility. Consequently, residual confounding cannot be excluded. The study did not include objective clinical indicators of diabetes management, such as glycated hemoglobin (HbA1c), fasting blood glucose, diabetes-related hospitalizations, or complication severity. The distribution of religious coping scores warrants consideration. Positive religious coping scores were highly concentrated at the upper end of the scale, whereas negative religious coping scores were concentrated at the lower end. The limited variability in negative religious coping may have reduced statistical power to detect associations with diabetes self-care and may partially explain the absence of significant findings for this dimension. Selection bias is also possible. Participants who agreed to participate may differ systematically from individuals who declined participation. The findings should also be interpreted within their cultural context. Saudi Arabia is a highly religious society in which religion is deeply integrated into social and cultural life. The prevalence, expression, and meaning of religious coping may differ substantially across populations with different religious traditions, levels of religiosity, or sociocultural environments. In addition, the recruitment strategy may have introduced selection bias. Participants were recruited from four purposively selected primary healthcare centers in Riyadh using nurse-facilitated invitations and an electronic questionnaire accessed via QR codes. Although this approach facilitated efficient and voluntary participation, it may have underrepresented individuals with limited digital literacy, reduced access to smartphones or electronic technologies, or lower willingness to complete electronic surveys.

## 5. Conclusions

In this cross-sectional study, positive religious coping was associated with higher diabetes self-care scores, whereas negative religious coping was not significantly associated with diabetes self-care. These findings suggest that positive religious coping may represent a relevant psychosocial correlate of diabetes self-management in this population. However, because of the cross-sectional design, no conclusions regarding causality can be drawn. Future longitudinal studies should incorporate objective clinical outcomes, including HbA1c, fasting blood glucose, diabetes-related complications, healthcare utilization, and diabetes-related hospitalizations, while also evaluating potential mediating and confounding factors such as self-efficacy, diabetes distress, health literacy, diabetes knowledge, depression, anxiety, social support, and access to care to better understand the pathways underlying these associations. Although positive religious coping may represent a relevant psychosocial correlate of diabetes self-care, its integration into diabetes education or behavioral interventions should be evaluated in well-designed prospective and interventional studies before being adopted in routine clinical practice.

## Figures and Tables

**Table 1:** Summary results of sociodemographic and clinical characteristics of participants.

Characteristic	N (%) or Mean ± SD
Age group	
18–24 years	23 (5.6)
25–34 years	60 (14.5)
35–44 years	90 (21.8)
45–54 years	121 (29.3)
55–65 years	65 (15.7)
>65 years	54 (13.1)
Gender	
Male	283 (68.5)
Female	130 (31.5)
Employment status	
Employed	236 (57.1)
Unemployed	88 (21.3)
Retired	65 (15.7)
Student	24 (5.8)
Marital status	
Married	202 (48.9)
Single	163 (39.5)
Widowed	33 (8.0)
Divorced	15 (3.6)
Educational level	
Below secondary	79 (19.1)
Secondary	129 (31.2)
Diploma	35 (8.5)
Bachelor or higher	170 (41.2)
Duration of diabetes	
<1 year	63 (15.3)
1–5 years	137 (33.2)
6–10 years	85 (20.6)
>10 years	128 (31.0)
Works in healthcare field	
Yes	69 (16.7)
No	344 (83.3)
Has chronic diseases	
Yes	273 (66.1)
No	140 (33.9)
Current treatment type	
Oral medications	90 (21.8)
Insulin	140 (33.9)
Oral medications + insulin	102 (24.7)
Diet only	81 (19.6)
Has diabetes complications	
Yes	111 (26.9)
No	302 (73.1)
Doctor visits per year	
<1 visit/year	57 (13.8)
1 visit/year	40 (9.7)
2 visits/year	114 (27.6)
3–4 visits/year	123 (29.8)
>4 visits/year	79 (19.1)
Positive religious coping score	3.72 ± 0.52
Negative religious coping score	1.74 ± 0.91
Total diabetes self-care score (days)	3.26 ± 1.44

**Table 2:** Associations between religious coping and diabetes self-care among adults with diabetes.

Covariates	Model 1 (Unadjusted Positive Religious Coping) β (95% CI)	Model 2 (Unadjusted Negative Religious Coping) β (95% CI)	Model 3 (Fully Adjusted Model) β (95% CI)
Positive religious coping	0.46 (0.22, 0.70)	—	0.44 (0.18, 0.70)
Negative religious coping	—	0.08 (−0.09, 0.25)	0.04 (−0.13, 0.22)
Age group (Ref: 18–24 years)			
25–34 years			0.24 (−1.05, 1.54)
35–44 years			0.21 (−1.03, 1.46)
45–54 years			0.10 (−1.15, 1.34)
55–65 years			0.43 (−0.89, 1.76)
>65 years			0.12 (−1.25, 1.48)
Gender (Ref: Male)			
Female			0.17 (−0.22, 0.55)
Employment status (Ref: Employed)			
Unemployed			−0.11 (−0.58, 0.36)
Retired			0.16 (−0.40, 0.72)
Student			0.19 (−1.10, 1.48)
Marital status (Ref: Married)			
Single			0.19 (−0.21, 0.58)
Widowed			0.16 (−0.49, 0.82)
Divorced			0.06 (−0.83, 0.94)
Educational level (Ref: Below secondary)			
Secondary			0.32 (−0.28, 0.92)
Diploma			0.22 (−0.58, 1.02)
Bachelor or higher			0.59 (−0.04, 1.23)
Duration of diabetes (Ref: <1 year)			
1–5 years			0.49 (−0.08, 1.07)
6–10 years			0.51 (−0.14, 1.17)
>10 years			0.44 (−0.21, 1.09)
Works in healthcare field (Ref: Yes)			
No			−0.19 (−0.72, 0.34)
Chronic disease (Ref: Yes)			
No			0.25 (−0.14, 0.63)
Treatment type (Ref: Oral medications)			
Insulin			0.23 (−0.16, 0.63)
Oral medications + insulin			0.58 (0.08, 1.09)
Diet only			0.25 (−0.28, 0.77)
Diabetes complications (Ref: Yes)			
No			−0.25 (−0.67, 0.18)
Doctor visits per year (Ref: <1 visit/year)			
1 visit/year			−0.09 (−0.76, 0.58)
2 visits/year			0.30 (−0.27, 0.86)
3–4 visits/year			0.33 (−0.25, 0.91)
>4 visits/year			0.62 (−0.03, 1.26)

Note: Model 1: Unadjusted association between positive religious coping and diabetes self-care. Model 2: Unadjusted association between negative religious coping and diabetes self-care. Model 3: Adjusted for positive religious coping, negative religious coping, age, sex, employment status, marital status, educational level, diabetes duration, healthcare occupation, chronic disease status, treatment type, diabetes complications, and physician visits.

## Data Availability

The data presented in this study are available on request from the first author due to they contain identifiable participant information and are subject to the privacy and confidentiality requirements of the Institutional Review Board of King Saud University.

## Supplementary Materials

The following supporting information can be downloaded at: https://www.mdpi.com/article/10.3390/healthcare14162440/s1, Figure S1. Correlation matrix of religious coping and diabetes self-care measures among adults with diabetes. Figure S2. Diagnostic plots for the fully adjusted linear regression model predicting diabetes self-care. Table S1. Internal Consistency Reliability of the Brief RCOPE Subscales. Table S2. Quantile Regression Models Examining Factors Associated with Diabetes Self Care Across the 25th, 50th, and 75th Percentiles of the Self Care Distribution. Table S3. Assessment of multicollinearity among independent variables in the fully adjusted regression model.
