# Supplementary material for: Religious Coping and Diabetes Self-Care Behaviors Among Adults with Diabetes in Saudi Arabia: A Cross Sectional Study
Source: Healthcare (Basel). 2026 Aug 7;14(16):2440. doi: 10.3390/healthcare14162440 (PMC13512128; doi:10.3390/healthcare14162440)
Supplement: Supplementary file 1 [file healthcare-14-02440-s001.zip › healthcare-4411646-supplementary.pdf]

Supplementary Materials

Religious Coping and Diabetes Self Care Behaviors Among Adults with Diabetes in Saudi Arabia: A Cross Sectional Study

Alya Alghamdi[1*], Majed M. Aljabri[1], Bandar S. Alharbi[1*]

[1]Community and Psychiatric Mental Health Nursing Department, College of Nursing, King Saud University, Riyadh 12375, Saudi Arabia

[*] Shared corresponding author (Email: aalgamdii@ksu.edu.sa; banalharbi@ksu.edu.sa)

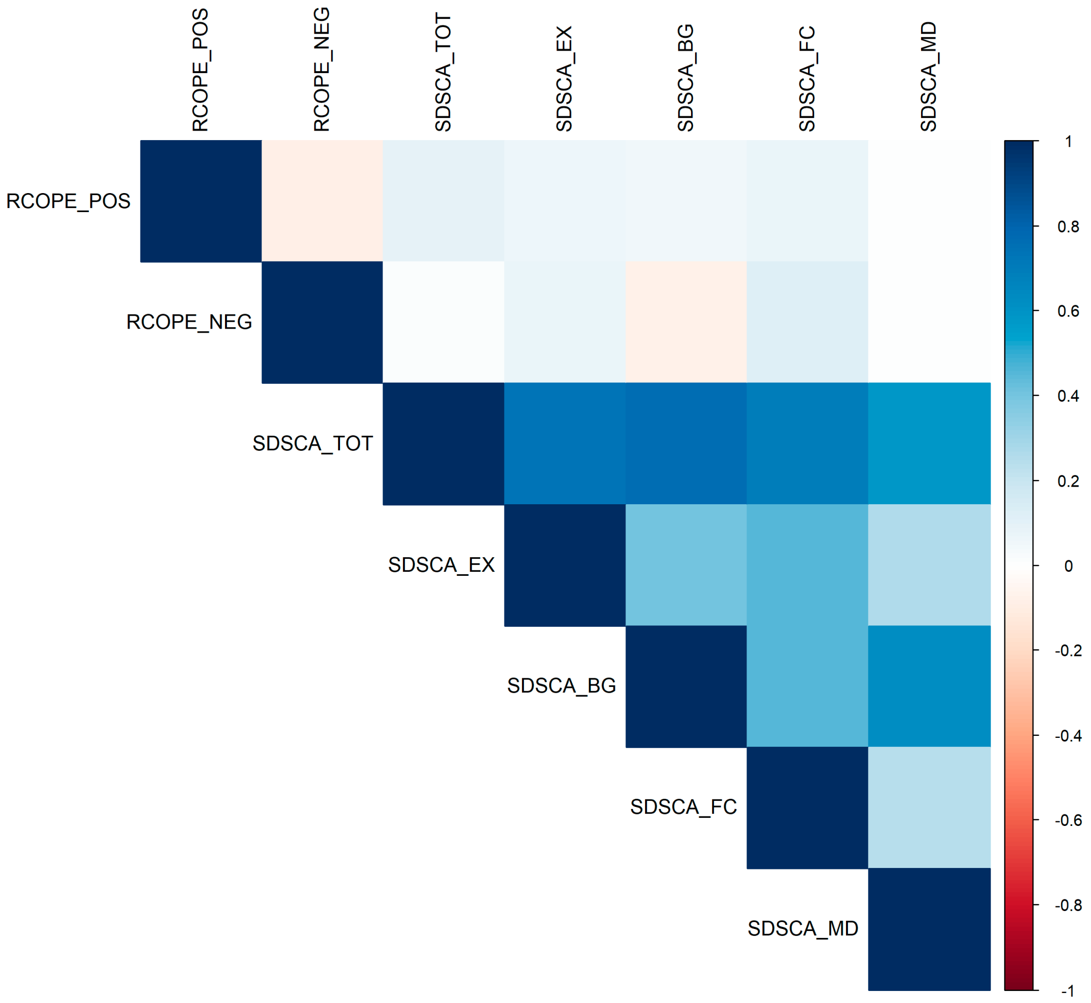

Figure S1. Correlation matrix of religious coping and diabetes self-care measures among adults with diabetes

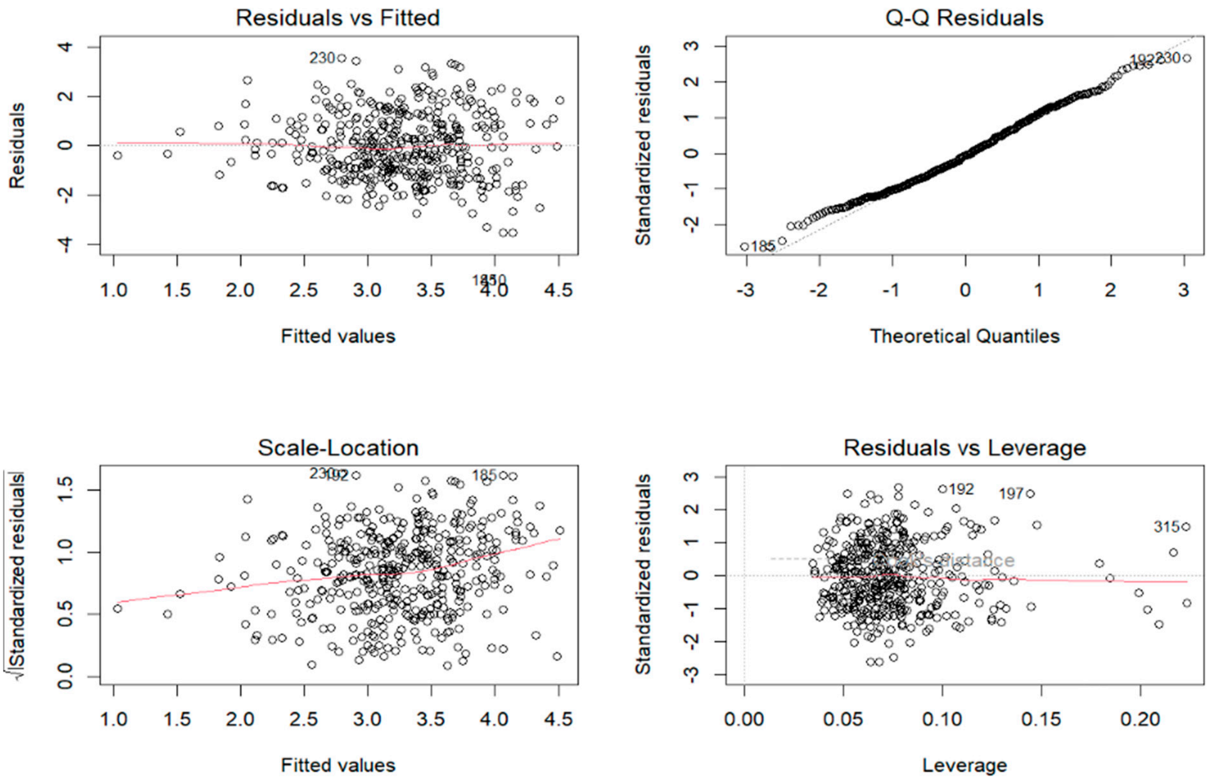

Figure S2. Diagnostic plots for the fully adjusted linear regression model predicting diabetes self-care

Table S1. Internal Consistency Reliability of the Brief RCOPE Subscales

Scale / Item	Mean ± SD	Cronbach's α if Item Deleted
Positive Religious Coping (7 items)	3.72 ± 0.52	Overall α = 0.92 (95% CI: 0.91–0.93)
RCOPE1	3.8 ± 0.57	0.91
RCOPE2	3.8 ± 0.57	0.90
RCOPE3	3.7 ± 0.69	0.92

RCOPE4	3.8 ± 0.58	0.91
RCOPE5	3.7 ± 0.69	0.92
RCOPE6	3.8 ± 0.59	0.90
RCOPE7	3.6 ± 0.66	0.91
Negative Religious Coping (7 items)	1.74 ± 0.91	Overall α = 0.92 (95% CI: 0.91–0.93)
RCOPE8	1.8 ± 1.18	0.92
RCOPE9	1.9 ± 1.15	0.91
RCOPE10	1.7 ± 1.09	0.91
RCOPE11	1.8 ± 1.11	0.91
RCOPE12	1.4 ± 0.91	0.91
RCOPE13	1.7 ± 1.07	0.91
RCOPE14	1.9 ± 1.13	0.92

Table S2. Quantile Regression Models Examining Factors Associated with Diabetes Self Care Across the 25th, 50th, and 75th Percentiles of the Self Care Distribution

Variable	τ = 0.25 β (95% CI)	τ = 0.50 β (95% CI)	τ = 0.75 β (95% CI)
Religious coping			
Positive religious coping	0.41 (0.10, 0.73)	0.50 (0.10, 0.90)	0.48 (0.10, 0.85)
Negative religious coping	−0.04 (−0.24, 0.16)	0.01 (−0.26, 0.27)	0.05 (−0.25, 0.35)
Age group (Ref: 18–24 years)			
25–34 years	0.12 (−1.30, 1.53)	0.79 (−1.11, 2.69)	0.28 (−1.90, 2.45)
35–44 years	0.25 (−1.13, 1.63)	1.01 (−0.80, 2.82)	0.23 (−1.89, 2.35)
45–54 years	0.12 (−1.24, 1.48)	0.70 (−1.12, 2.51)	0.05 (−1.98, 2.09)
55–65 years	0.45 (−0.98, 1.89)	1.25 (−0.77, 3.28)	0.29 (−1.87, 2.45)
>65 years	0.59 (−0.98, 2.15)	1.25 (−0.81, 3.30)	−0.45 (−2.65, 1.74)
Gender (Ref: Male)			
Female	0.21 (−0.26, 0.68)	0.12 (−0.41, 0.66)	0.28 (−0.40, 0.97)
Employment status (Ref: Employed)			

Unemployed	0.24 (−0.36, 0.83)	−0.19 (−0.90, 0.52)	−0.10 (−0.90, 0.71)
Retired	−0.23 (−0.92, 0.46)	0.05 (−0.85, 0.95)	0.51 (−0.44, 1.45)
Student	0.21 (−1.24, 1.65)	0.53 (−1.30, 2.35)	0.00 (−2.05, 2.04)
Marital status (Ref: Married)			
Single	0.28 (−0.22, 0.78)	0.43 (−0.14, 0.99)	0.12 (−0.50, 0.74)
Widowed	−0.14 (−1.06, 0.78)	−0.02 (−1.06, 1.01)	0.56 (−0.37, 1.48)
Divorced	−0.24 (−1.28, 0.80)	−0.39 (−1.79, 1.01)	−0.12 (−1.78, 1.55)
Educational level (Ref: Below secondary)			
Secondary	0.54 (−0.19, 1.27)	0.09 (−0.93, 1.12)	0.24 (−0.82, 1.30)
Diploma	0.10 (−0.79, 1.00)	−0.09 (−1.21, 1.03)	−0.40 (−1.81, 1.01)
Bachelor or higher	0.72 (−0.02, 1.46)	0.55 (−0.43, 1.53)	0.48 (−0.63, 1.59)
Duration of diabetes (Ref: <1 year)			
1–5 years	−0.06 (−0.72, 0.61)	0.63 (−0.23, 1.50)	0.84 (0.03, 1.66)
6–10 years	0.01 (−0.75, 0.78)	0.67 (−0.24, 1.59)	1.11 (0.18, 2.05)
>10 years	−0.04 (−0.78, 0.70)	0.37 (−0.52, 1.26)	0.79 (−0.18, 1.77)
Works in healthcare field (Ref: Yes)			
No	0.23 (−0.58, 1.04)	0.08 (−0.68, 0.83)	−0.37 (−1.19, 0.45)
Chronic disease (Ref: Yes)			
No	0.06 (−0.41, 0.54)	0.31 (−0.24, 0.87)	0.46 (−0.08, 1.01)
Treatment type (Ref: Oral medications)			
Insulin	0.05 (−0.39, 0.49)	0.29 (−0.28, 0.86)	0.14 (−0.49, 0.78)
Oral medications + insulin	0.21 (−0.33, 0.75)	0.67 (−0.15, 1.48)	0.65 (−0.19, 1.49)
Diet only	0.22 (−0.54, 0.99)	0.17 (−0.66, 1.00)	0.62 (−0.20, 1.45)
Diabetes complications (Ref: Yes)			
No	−0.18 (−0.71, 0.35)	−0.25 (−0.91, 0.42)	−0.37 (−0.99, 0.24)
Doctor visits per year (Ref: <1 visit/year)			
1 visit/year	0.06 (−0.78, 0.90)	−0.39 (−1.54, 0.76)	−0.01 (−1.11, 1.09)

2 visits/year	0.55 (−0.08, 1.18)	0.34 (−0.66, 1.33)	0.32 (−0.65, 1.28)
3–4 visits/year	0.54 (−0.15, 1.22)	0.19 (−0.86, 1.24)	0.56 (−0.40, 1.51)
>4 visits/year	0.76 (0.07, 1.46)	0.74 (−0.36, 1.83)	0.80 (−0.34, 1.94)

Table S3. Assessment of multicollinearity among independent variables in the fully adjusted regression model

Variable	GVIF	df	Adjusted GVIF†
Positive religious coping	1.12	1	1.06
Negative religious coping	1.12	1	1.06
Age group	15.98	5	1.32
Gender	1.65	1	1.28
Employment status	12.99	3	1.53
Marital status	2.89	3	1.19
Educational level	3.38	3	1.23
Duration of diabetes	3.21	3	1.21
Healthcare worker status	1.49	1	1.22
Chronic disease status	1.39	1	1.18
Treatment type	2.38	3	1.16
Diabetes complications	1.54	1	1.24
Doctor visits per year	1.95	4	1.09
